# Allocating operating room time in orthopaedic trauma: a survey in medical ethics

**DOI:** 10.1007/s10389-024-02232-z

**Published:** 2024-04-06

**Authors:** Mary-Katherine Lynch, Gabriella Rivas, Mathew J. Gregoski, Langdon Hartsock, Kristoff Reid

**Affiliations:** 1 Department of Orthopaedics and Physical Medicine, Medical University of South Carolina, 96 Jonathan Lucas St, CSB 708, Charleston, SC 29425 USA; 2 Department of Public Health Sciences, Medical University of South Carolina, 135 Cannon St, Suite 303, Charleston, SC 29425 USA

**Keywords:** Operating room time, Allocation of limited medical resources, Trauma orthopaedics, Case priority, Medical ethics

## Abstract

**Introduction:**

Allocation of operating room time is a challenging dilemma that surgeons frequently confront. When deciding upon a daily caseload, the surgeon may consider clinical, logistical, and social factors. Although previous studies have outlined guiding principles, there is no universal algorithm for prioritizing surgical patients. Our study aims to learn which factors orthopaedic trauma surgeons use when determining case priority.

**Methods:**

A survey regarding the allocation of operating room time was administered to orthopaedic trauma surgeons from the community and members of the Southeastern Fracture Consortium. Questions included a list of characteristics and a series of theoretical case scenarios to be ranked according to perceived priority.

**Results:**

Of the participating surgeons, 92% practiced at an academic center and 89.7% at a level 1 trauma center. Of the case characteristics, “severity of orthopaedic problem” was most frequently ranked as most important versus “social pressure from family members,” which was most frequently ranked as least important in case priority. The coefficient of concordance among respondents was 0.427 for individual case characteristics versus 0.287 for the theoretical scenarios. The average rate of agreement among respondents was 31.9 ± 19% for individual factors versus 36.3 ± 8.9% in the clinical vignettes.

**Conclusions:**

A consensus exists regarding severity of the presenting orthopaedic problem being the most important factor when considering case priority. The lower agreement in the clinical vignettes indicates a strong interplay between the multiple factors in a case. Survey commentary suggests that outside factors – training, experience, politics, the team available – also play a role in a surgeon’s decision on case priority.

**Level of evidence:** IV.

**Supplementary Information:**

The online version contains supplementary material available at 10.1007/s10389-024-02232-z.

## Introduction

Allocation of operating room time, a limited medical resource, is a challenging dilemma in orthopaedic trauma. When deciding upon a daily caseload, the surgeon may consider clinical, logistical, and social factors. While there is no universal algorithm for prioritizing surgical patients, authors have previously outlined guiding principles to aid in decision making. In a 2009 paper, Persad et al. identified four general principles that have been used to guide allocation of scarce resources in military and medical operations. They include (1) a first-come, first-served mentality, (2) prioritization of the sickest, (3) maximization of number of people helped, and (4) promoting social usefulness and honoring reciprocity (Persad et al. 2009).

Upon review of these principles and the implications of applying them to a list of patients in need of orthopaedic surgical care, it becomes clear that they cannot all be followed simultaneously. Kelly et al. expanded upon Persad’s principles and proposed surgeon and institution level recommendations related to trauma operating room allocation, including development of multidisciplinary panels to regularly review institutional practices and timely communication of delays to patients (Kelly et al. 2022). However, actual decision making for case order remains an ongoing dilemma for orthopaedic trauma surgeons.

Despite the medical ethical principles advanced by Persad and Kelly, there seems to be little research investigating the actual application of these ideals to real life scenarios. There has been some recent interest in studying prioritization of backlogged elective cases in the setting of the COVID-19 pandemic, but no studies in the trauma population (Scott 2022). As such, the purpose of this study was to learn more about the factors orthopaedic trauma surgeons use to make decisions on case priority, and to study how those factors would be applied to realistic case scenarios. We hypothesized that there would be high consensus when ranking independent factors, but more discordance when considering complex cases. 

## Methods

A 30-question survey was created and administered to orthopaedic trauma surgeons from January to March 2023. The survey was developed by one of the authors (MKL), with the remaining study authors reviewing the survey and incorporating edits into the final instrument. Although requested, it was determined that institutional review board oversight was not required. The survey was distributed among trauma surgeon members of the Southeastern Fracture Consortium and community trauma surgeons known to or near our institution. The survey contained only de-identified questions and was managed on the secure REDCap web platform.

The survey comprised questions that inquired about the allocation of operating room time and aimed toward gaining the respondents’ insight into the factors used by orthopaedic trauma surgeons when deciding the priority of their cases. Participants were first asked to consider a list of 11 factors and rank them by importance in relation to case prioritization. Following factor ranking, participants were asked to consider and rank a list of six nuanced patient cases and rank them by urgency. Additionally, the survey collected relevant demographics pertaining to the respondent’s role, subspecialty training, and the academic nature and trauma center level of their practicing institution. A box for free text response was also provided for respondents to explain their reasoning or add context. A copy of the instrument can be found in Appendix 1.

Data collection was completed on Microsoft Excel (Washington, USA) and statistical analysis was performed on IBM SPSS Statistic v28 (Armonk, NY). Descriptive statistics and Friedman’s analyses of variance (ANOVA) were used as appropriate. Statistical significance was set at an alpha of *p* < 0.05.

## Results

### Demographics

A total of 53 orthopaedic surgeons received the survey link, out of which 73% of responses (39/53) were included due to completeness. Ninety-two percent of responses came from surgeons practicing at an academic center; 89.7% reported working at a level 1 trauma center, 7.7% at a level 2 trauma center, and 2.6% at either a level 3 trauma center or higher or a community hospital. Additionally, 89.7% of respondents identified as “trauma” surgeons, 5.1% as “general,” and 5.1% as “arthroplasty” surgeons (Table 1). Due to the lack of homogeneity among the sample for trauma level center and surgeon type, between group analyses were not completed.

**Table 1:** Demographics of survey participants

	Number (n)	Percentage (%)
Type of training center		
Academic center	36	92%
Non-academic center	3	8%
Level of trauma center		
Level 1	35	89.7%
Level 2	3	7.7%
Level 3 or community	1	2.6%
Orthopaedic subspecialty		
Trauma	35	89.7%
General	2	5.1%
Arthroplasty/joints	2	5.1%

### Ranking of case characteristics

The rankings of the 11 independent case characteristics were examined using a Friedman’s ANOVA with Kendall’s W coefficient as a measure of effect size. Results revealed a significant difference among characteristics χ^2^(10) = 166.55 (*p* < 0.001) with Kendall’s coefficient of concordance = 0.427, indicating moderate agreement among responders. Out of the 11 case characteristics included in the survey, “severity of orthopaedic problem” was most frequently ranked as the most important when considering case priority (mean rank, 1.78). Respondents then identified “number of days already waiting for surgery” (mean rank, 4.04), “severity of medical comorbidities” (mean rank, 4.32), and “quality of reduction/current immobilization” (mean rank, 4.63) as the next most important factors to consider case prioritization. These were followed by “skillset/scope of practice of weekend call attending” with a mean rank of 5.56. At the bottom of the list, “social pressure from family members” was most frequently ranked as the least important characteristic playing a role in case priority (mean rank, 8.87). “Personal/social connection to patient out of the hospital” (mean rank, 8.01) and “patients is from out of town waiting on this surgery” (mean rank, 8.28) were similarly considered less important. Table 2 shows the mean rankings for each characteristic, demonstrating a significant skew toward “orthopaedic problem” being ranked first. The average percent of surgeons who agreed on rankings was 31.9 ± 19%.

**Table 2:** Mean rankings for each independent case characteristic

Factor	Mean ranking
Severity of orthopaedic problem	1.78
Number of days already waiting for surgery	4.04
Severity of medical comorbidities	4.32
Quality of reduction/current immobilization	4.63
Skillset/scope of practice of weekend call attending	5.56
Age	6.37
Surgery is the last barrier to discharge	6.79
Request from primary team to prioritize the case	7.33
Personal/social connection to patient out of the hospital	8.01
Patient is from out of town waiting on this surgery	8.28
Social pressure from family members	8.87

### Clinical vignettes

A total of six theoretical clinical scenarios were provided in the survey, and the respondents were asked to review each case and assign a value, 1–6, indicating the priority of adding on the case as the last surgical procedure on a Friday afternoon, assuming all emergent overnight cases have already been addressed; 71.8% of respondents (28/39) completed the vignette section. The six vignettes were examined using a Friedman’s ANOVA with Kendall’s W as a measure of effect size. Results revealed a significant difference among characteristics χ^2^(10) = 40.119 (*p* < 0.001) with Kendall’s coefficient of concordance = 0.287, indicating fair agreement among responders. Patient D, an 80-year-old female presenting with a periprosthetic distal femur fracture status-post fall while transferring, was most commonly ranked as the most likely case to be addressed, with a mean rank of 1.82. Ranked the second most important was patient C, a morbidly obese 65-year-old male who sustained a closed tri-malleolar ankle fracture, currently with inadequate reduction (mean rank, 2.80). Conversely, two cases were most commonly ranked as the least likely case to be added on to the schedule. Patient F, a 48-year-old female presenting with a closed both bone forearm fracture and adequate reduction in a sugar tong splint, was ranked last, with a mean rank of 4.39. Patient A, a 90-year-old female with a closed comminuted intraarticular distal humerus fracture who will not be able to have surgery over the long weekend, was closely ranked as less likely (mean rank, 4.23). The average percent of surgeons who agreed on case priority rankings was 36.3% (SD ± 8.9%). The full theoretical clinical vignettes can be found within the survey in Appendix 1.

## Discussion

This survey study of orthopaedic surgeons revealed that there is a high consensus among surgeons regarding the most and least important factors to consider when allocating operating room time. When selecting from a simple list of factors, 84.6% of respondents agreed that severity of orthopaedic problem was the most important factor. This selection aligns with Persad’s guiding principle of prioritarianism or prioritizing the sickest first (Persad et al. 2009). The factors that followed closely behind, severity of medical comorbidities and number of days waiting for surgery, also touch on Persad’s principles of prioritarianism and first come first serve, respectively (Persad et al. 2009). Toward the end of the list, personal or social connection to the patient, as well as social pressure from family members, were commonly ranked as the least important, suggesting that among the respondents, patient factors were viewed as more important than honoring social relationships.

In accordance with our hypothesis, only 53.6% of respondents agreed on the case of highest importance when asked to consider real-life scenarios, even though they had largely agreed that severity of orthopaedic problem was the most important decision-making factor. This is demonstrated by the lower coefficient of concordance seen among the vignettes when compared to the case characteristics. It is possible that the respondents, despite having similar training, do not agree on severity of orthopaedic problem. Additionally, it is most likely that the introduction of numerous competing factors in a single case added nuances and complexities that complicated decision making, and thus introduced discordance between the respondents.

Patient D, which was most frequently ranked as the most likely case to be addressed, featured an interplay of the following individual factors: a severe orthopaedic problem, geriatric status, and high social pressures from family and hospital members. Patient C, who was ranked as the second most likely case to be addressed, featured a medically complex patient with a difficult reduction. The selection of these two cases correlated with the rankings of individual factors, highlighting a prioritization of the severity of the patient’s orthopaedic problem. Many respondents indicated considering the femoral fracture to be the most orthopaedically severe among all cases presented. However, selection of the scenario with the strongest social pressures contradicted the individual rankings which suggested that social influences were not highly regarded in the decision-making process of operating room time allocation.

To better understand the true interplay between multiple factors within a single scenario, the survey also provided a free text block for respondents to offer insight as to what factors influenced their decision to rank a case #1. Those that chose the periprosthetic distal femur fracture (case D) as the case to add on, which comprised 56% of respondents, cited a variety of reasons for their decision. Respondents mentioned geriatric status, high morbidity/mortality risk, and potential benefits of long bone fracture fixation but also cited inherent social pressures of the patient’s relation to a prominent donor and the direct communication from administration. This suggests that despite surgeons’ best efforts to ignore non-medical influences, they may enter in some part into decision making. However, it is important to note that given that patient D featured both a high-risk fracture and a strong social pressure, these two factors may be confounding the perceived importance of the other within the same scenario.

Of note, the percentage of respondents who completed the survey in its entirety was 71.8% (28/39), meaning 11 surgeons ranked the list of factors, but chose not to rank the clinical vignettes. While several things may have contributed to this attrition – i.e., time burden, viewing the vignettes on a phone screen vs computer etc. – the mental load of making these decisions cannot be overlooked. This is important to consider, as orthopaedic trauma surgeons grapple with these decisions on a daily basis, likely adding to work-related mental fatigue.

In the proposed scenario, in which a surgeon is selecting an additional case to add on a Friday afternoon, the physiological and psychological comfort of the operating surgeon and surgical team should be considered alongside Persad’s guiding principles (McLean et al. 2021; Persad et al. 2009). The survey free text blocks demonstrate the variety of surgeon approaches to this issue. Some respondents indicated selecting a “quick and easy case” while others preferred to “stay late and get the work done.” Although quite different, both approaches incorporate Kelly’s fifth guiding principle: efficiency–flexibility–resilience. The complexities behind the dilemma of OR allocation require above all a system that is efficient in its care, flexible to accommodate unforeseen circumstances, and resilient in its focus on optimal surgical care delivery (Kelly et al. 2022).

The dilemma of case order has previously been studied in both general and specialty surgery. A survey of ophthalmic surgeons indicated a surgeon tendency to schedule the most straight forward cases at the beginning of the day and reserve the last slots of the day for more complex cases. They concluded that surgeon comfort and dexterity may be at more optimal levels for complex cases at the end of the day, and the complexity of cases should be used as a guiding criterion for case order. Likewise, multiple studies have previously demonstrated longer operating times during the first cases of the day, and even substantial differences in case duration with each subsequent position in the order list (Gupta and Taravati 2015; Lavelle et al. 2017; Li et al. 2018; Pike et al. 2018). Further, Pike et al. reported additional accrued benefits when the same procedure was performed repeatedly and saw that switches in procedures resulted in an increase in operating time of 6.5% (Pike et al. 2018). Given the cognitive and technical requirements of surgery, these results introduce the concept of a “warm-up” phenomenon (Lavelle et al. 2017). While our study addressed case priority, the survey did not include similarity of proposed case to previously completed case as a factor and did not introduce this concept in the vignettes, which may be worth further investigation.

Recently, the COVID-19 pandemic served as a clear example of the dilemma of allocating limited medical resources. Despite the ongoing pandemic and curtailing of elective surgical procedures, hospitals faced the need to continue with urgent and emergent surgical cases such as those of hospitalized or trauma patients. Some hospitals applied a scoring system integrating procedure, disease, and patient factors for cases designated as “medically necessary, time-sensitive,” which incorporated ethical and efficiency considerations, limited the risk of transmission to healthcare providers, served as a triage process, and offloaded the ethical pressures from the surgical providers (Prachand et al. 2020). Other institutions established multidisciplinary committees to oversee operating room time and review surgeon requests for urgent cases, according to severity and urgency of the patient’s condition (Tanzer et al. 2020). Regardless of the chosen approach, the COVID-19 pandemic demanded an effective application of the aforementioned guiding principles for case prioritization. While the pandemic highlighted the ethical dilemma of allocating OR time, these quandaries have long been present in the orthopaedic trauma community and as the pandemic committees dissolve, these decisions will once again be the responsibility of the surgeon.

There are several limitations to our study. The utilization of a survey instrument introduces both recall bias and the possibility of a Hawthorne effect on survey respondents. Given the ethical implications in allocating medical resources, we cannot exclude the possibility that survey responses were biased toward what surgeons perceive to be most ethical rather than what is true in their practice. This effect is likely less prevalent in the hypothetical scenarios, weakened by the interplay between factors. Future studies should consider a discrete choice experiment approach to better discern tendencies and the weights of such when considering cases with multiple influencing factors. In addition, the free text comments revealed additional case considerations that might also be of value to surgeons that were not explicitly addressed in the independent factors or case scenarios. These might include procedure length as perhaps surgeons would have prioritized short case length in order to clear two cases from the board in alignment with Persads guiding principle of maximization, or similarity of proposed case to previously completed case as discussed above.

## Conclusion

As demonstrated in this survey, there is no universal approach to the OR allocation dilemma. While orthopaedic trauma surgeons appear to be in consensus on the independent factors that drive case priority, the decreased agreement in clinical vignettes demonstrates the nuances and complexities added by the combination of competing case characteristics. Survey commentary suggests that outside factors such as training, experience, politics, and the team available at the practicing institution also play a role in a surgeon’s decision on case priority. The daily battle of OR allocation should be approached by surgeons with consideration of both the proposed ethical guiding principles as well as patient-specific characteristics that can impact surgical performance.

## Supplementary Information

Below is the link to the electronic supplementary material.

Supplementary file1 (DOCX 30 KB)

## Data Availability

Data is not publicly available but can be made available upon request to the authors.
