# Supplementary material for: Allocating operating room time in orthopaedic trauma: a survey in medical ethics
Source: J Public Health (Berl). 2024 Apr 6;34(1):9–13. doi: 10.1007/s10389-024-02232-z (PMC12823663; doi:10.1007/s10389-024-02232-z)
Supplement: Supplementary file 1 — Supplementary file1 (DOCX 30 KB) [file 10389_2024_2232_MOESM1_ESM.docx]

**A Study in Surgical Ethics**

What factors are most influential when considering allocation of orthopaedic trauma operating room time?

**Background**: Allocation of operating room time, a limited medical resource, is a challenging dilemma which orthopaedic trauma surgeons confront on a daily basis. When deciding upon a daily caseload, the surgeon may consider clinical, logistical and social factors. While Persad et. al outlined four “guiding principles” to help inform these decisions, there is no universal algorithm or scoring system by which we prioritize surgical patients. We are interested in learning more about what factors orthopaedic trauma surgeons use when deciding what cases to prioritize and which to roll over to the next day.

You are being asked to participate in this research study because of your role in patient care in the operating room setting. Your participation in this survey is completely voluntary.

This survey should take about 5-10 minutes to complete.

Thank you for your participation!

**Do you practice at an academic or non-academic institution?**

___ Academic

___ Non-Academic

**Is your institution a …?**

___ Level 1 Trauma Center

___ Level 2 Trauma Center

___ Level 3+ Trauma Center / Community Hospital

**Orthopaedic Subspecialty**: ____________________

**Role**:

___ Surgeon/Provider

___ Resident

___ Physician Assistant

___ Other

**If “Other,” please explain**: ____________________

**Please rank the following factors by importance when considering case priority.**

1 = most important

11 = least important.

___ Age

___ Severity of orthopedic problem

___ Severity of medical comorbidities

___ Quality of reduction/current immobilization

___ Number of days already waiting for surgery

___ Surgery is the last barrier to discharge

___ Patient is from out of town waiting on this surgery

___ Skillset/scope of practice of weekend call attending

___ Social pressure from family members

___ Request from primary team to prioritize the case

___ Personal/social connection to patient out of the hospital

**For the next exercise, read the following 5 clinical vignettes and imagine you have time to complete one additional case on a Friday afternoon.**

**You have treated all of the open fractures and hip fractures that came in overnight. Please rank the cases by priority.**

1= you would take as your final case

6= you would feel most comfortable rolling this case till tomorrow.

___ Patient A is an 90yo female s/p ground level fall with a closed comminuted intraarticular distal humerus fracture. She is medically cleared for surgery and has been admitted for one day. The covering attending over the long holiday weekend is a F+A specialist who does not feel comfortable tackling a comminuted distal humerus. If you do not operate today, she will remain inpatient waiting on OR for 5 potentially avoidable days.

___ Patient B is a 30yo otherwise healthy male who presents with left tri-malleolar ankle and numerous facial fractures fracture following fall on boat while vacationing in your city. The care of his facial fractures is complete. He has been NPO for the past three days waiting for surgery, and has a strong preference to have surgery at your facility as he has no established orthopaedic provider at home. He is notably frustrated on rounds when you explain that due to urgent cases (hip fractures, open fractures) he may not be able to have surgery today.

___ Patient C is a 65yo morbidly obese medically complex male who sustained a closed tri-malleolar ankle fracture falling down the stairs. He will likely require prolonged admission for continued care of his medical comorbidities. Due to body habitus, multiple reduction attempts fail to achieve a stable ankle mortise, and the post reduction x-ray taken yesterday demonstrates possible point loading of the talar dome.

___ Patient D is an 80 year old female who presents with periprosthetic distal femur fracture following fall while transferring from wheel chair to car. She has required several days of medical stabilization before being deemed appropriate for surgery yesterday. Since admission, you have received calls from both of her physician children, as well as the patient liaison service who reminds you that her family is prominent donor. At the start of the day, your chair mentions that her family reached out to him last night to ensure she was getting appropriate orthopaedic care.

­­___ Patient E is a 34yo polysubstance abuser who presented to the ED after being found down. On hospital day 3, he starts to complain of bilateral hip pain. During admission, he has been borderline febrile, a mildly tachycardic to ~108. His inflammatory markers are elevated. On exam, he does not tolerate ROM of R hip. There is no effusion on imaging, and interventional radiology is unable to obtain a sample with attempted aspiration. The primary team has eliminated all other sources of infection, and feels strongly that you need to wash out the hip.

___ Patient F is a 48yo female who presented to the ED with a closed both bone forearm fracture s/p fall onto the outstretched arm. She has no significant PMH, and is appropriately immobilized in a sugar tong splint, with her arm elevated. There is no concern for compartment syndrome. When you round on her, you both realize that she is a coach at your children’s tennis camp, and she has been very understanding of your late pickup times all summer. She has been NPO today, and is waiting only for surgery.

**For the case ranked as #1, please explain why you ranked it so**: ____________________________________________________________________________________________________________________________________________________________

**For the case ranked #6, please explain why you ranked it so**: ____________________________________________________________________________________________________________________________________________________________

**Thank you for your participation! If there are other factors we’ve missed or you have additional comments, please let us know below**: ____________________________________________________________________________________________________________________________________________________________
